# Self-assembly behaviors of perylene- and naphthalene-crown macrocycle conjugates in aqueous medium

**DOI:** 10.3762/bjoc.15.117

**Published:** 2019-06-03

**Authors:** Xin Shen, Bo Li, Tiezheng Pan, Jianfeng Wu, Yangxin Wang, Jie Shang, Yan Ge, Lin Jin, Zhenhui Qi

**Affiliations:** 1 Sino-German Joint Research Lab for Space Biomaterials and Translational Technology, School of Life Sciences, Northwestern Polytechnical University, Xi’an, Shaanxi 710072, P. R. China; 2 Institute of Biomedical Materials & Engineering (IBME), Northwestern Polytechnical University, Xi’an, Shaanxi 710072, P. R. China

**Keywords:** aqueous medium, crown ether, macrocycle, perylene diimide derivative, self-assembly

## Abstract

The synthesis of conjugates of perylene diimide (PDI) and naphthalene diimide (NDI) modified with two benzo-21-crown-7 ethers (B21C7) are herein described. Their self-assembly behavior in various solvents was investigated particularly in aqueous medium, due to the recently discovered hydrophilic properties of B21C7 crown macrocycle. An unexpected fluorescence quenching phenomenon was observed in the PDI-B21C7 macrocycle conjugate in chloroform. The detailed UV–vis absorption and fluorescence spectra of these PDI/NDI derivatives in different solvents as well as their morphologies were investigated.

## Introduction

Owing to their unique physicochemical properties, self-assemblies have been recognized as a type of exciting nanomaterials with tremendous potential for research and commercial applications [1–6]. Thanks to the development of supramolecular chemistry, the ordered structures could be obtained through programmed self-assembly coupled with covalent chemical synthesis [7]. In fact, supramolecular technology has shown great importance in various kinds of functional materials, including natural protein complexes [8–10], hydrogels [11–13], carbon-based materials [14–17], self-healing materials [18–23], and composite materials [24–26]. Moreover, several new proof-of-concept applications of supramolecular assemblies are also of increasing interest, specifically in the fields of energy generation and storage [27], water treatment and environmental remediation [28–29], and healthcare and biomedical engineering [30–32].

Nowadays, as the research in supramolecular chemistry is expanding into the aqueous realm, a systematic understanding of the interaction relationship of supramolecular self-assemblies and aqueous medium is becoming increasingly important [33–36]. As the first generation of macrocyclic hosts, crown ethers, have been widely used as building blocks for supramolecular assemblies in the past two decades [37–53], mainly in the fabrication of crown ether-based threaded or interlocked assemblies in organic solvents [54]. However, we recently found that benzo-21-crown-7 (B21C7) displays impressive high water solubility in comparison with other macrocyclic hosts [55]. Moreover, the conjugation of B21C7s to the well-known supramolecular BTA core leads to the observation of special topological effects on ion selectivity in water [56]. Following this water-centered view, we further explored a new type of supramolecular polymeric adhesive based on B21C7 derived low–molecular weight (LMW) monomer, in which the water molecules serve as essential co-monomers to the polymerization process, rather than as solvent [57]. Compared with their linear glycol chain counterparts, B21C7 shows great potential to be an easy-to-accessed building block to probe the non-covalent interactions and chemical transformations influenced by water molecules.

Perylene diimide (PDI) and naphthalene diimide (NDI) are polycyclic aromatic chromophores widely used in the dye industry and have been applied as advanced materials in the fields of fluorescence labeling [58–59], organic semiconducting devices [60–61], and light harvesting [62–65]. PDI and NDI units have been extensively incorporated into functional supramolecular architectures through hydrogen bonding and metal ion coordination in nonpolar solvents [66–68]. Compared with NDI, PDI has more aromatic rings to generate stronger intermolecular π−π stacking, leading to molecular aggregates more easily, and these aggregates or supramolecular assemblies can give rise to desirable properties, such as charge transport and photophysical properties [69–74]. While the morphologies of these aggregates were greatly influenced by solvents, molecular structures and shapes, as well as the fraction of hydrophilic and/or hydrophobic parts [75–81]. By far, little was known to the self-assembly behaviors of B21C7 crown macrocycle functionalized PDIs/NDIs in aqueous solution, even though there have been several reports about crown ether-functionalized PDIs or NDIs, which mostly focused on their interactions with metal ions [82–85]. Herein, we attempted to combine these two classical organic moieties and crown macrocycle together and reported the PDI- and NDI-B21C7 conjugates. Their self-assemblies behaviors in aqueous medium were thus probed.

## Results and Discussion

**Scheme 1:**
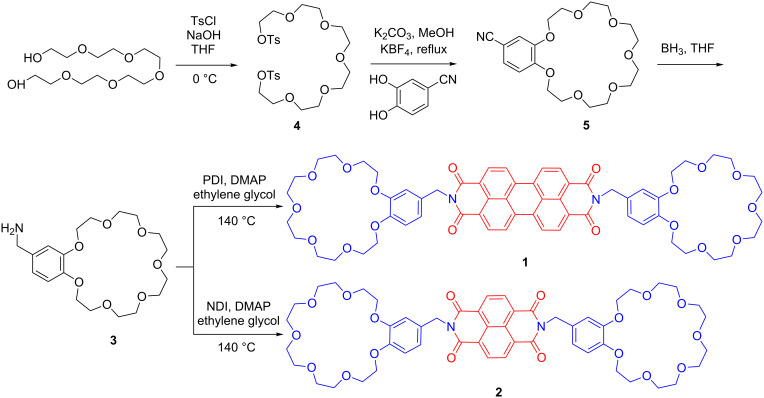
Synthetic route of perylene- and naphthalene-crown macrocycle conjugates **1** and **2**.

The synthetic route toward compounds **1** and **2** is shown in Scheme 1. Initially, the water-soluble crown macrocycle **3** was synthesized according to reported protocols, by using potassium cations as template [86–87]. The primary amine group in **3** offers diverse reaction pathways to design crown ethers appended LMW gelators, dendrimers and polymers [88–92]. In order to conjugate B21C7 onto PDI, compound **3** and 4-dimethylaminopyridine (DMAP) were dissolved in ethylene glycol, then 3,4,9,10-perylenetetracarboxylic dianhydride (PDI) was charged and suspended in the solution. The mixture was heated to 140 °C and stirred overnight. After cooling down to room temperature, the reaction mixture was dissolved in CH_2_Cl_2_. The solution was washed by diluted hydrochloric acid (0.05 M) and saturated brine, respectively. After the drying process, compound **1** was obtained with moderate yield. Compound **2** was prepared via a similar method except that NDI was used instead of PDI. All the final compounds were carefully characterized by ^1^H and ^13^C NMR spectroscopy and electrospray ionization mass spectroscopy (ESI-MS, Supporting Information File 1, Figures S1–S8).

**Figure 1:**
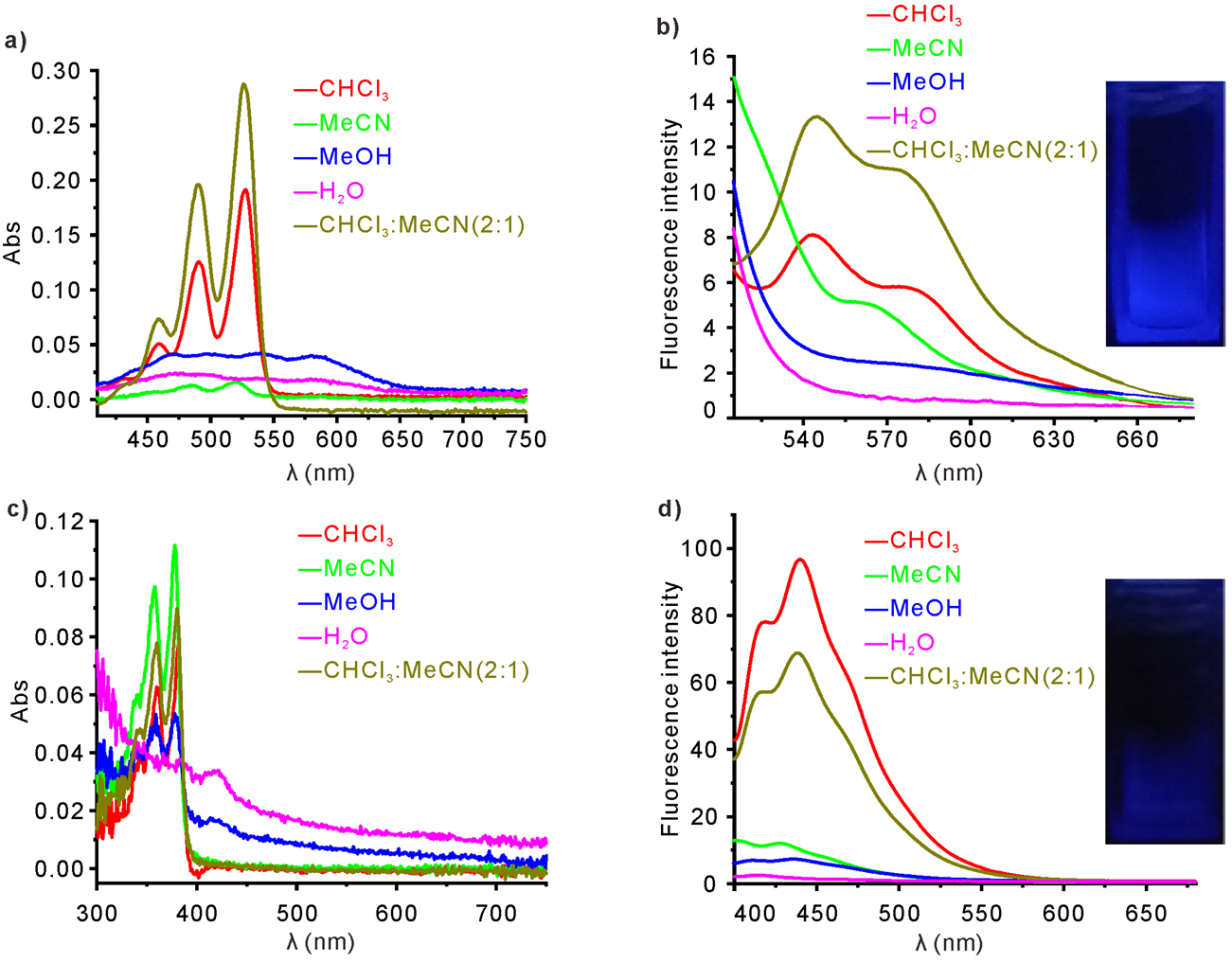
(a) UV–vis absorption spectra and (b) fluorescence spectra (λ_ex_ = 490 nm) of **1** in different solvents (inset: photograph of **1** in CHCl_3_ irradiated under 365 nm); (c) UV–vis absorption spectra and (d) fluorescence spectra (λ_ex_ = 360 nm) of **2** in different solvents (inset: photograph of **2** in CHCl_3_ irradiated under 365 nm). The concentration of **1** or **2** in the solutions is 5.0 × 10^−6^ M. All the measurements were performed at room temperature.

UV–vis absorption and fluorescence spectra of **1** in different solvents, including CHCl_3_, MeCN, MeOH, H_2_O, and mixed solvent CHCl_3_/MeCN (v/v = 2:1), were recorded to investigate its self-assembly behavior. Figure 1a shows the UV–vis absorption spectra of **1** in solvents with different polarity. The spectrum of **1** in the less polar solvent CHCl_3_ shows a well-resolved vibronic structure (0–0, 0–1, and 0–2 transitions of PDI molecules) with three characteristic absorption bands at 530, 485, and 469 nm, respectively [67]. However, the ratio of intensities of the bands derived from 0–0 and 0–1 transitions is around 1.53, indicating that **1** in CHCl_3_ seems do not only exist in monomeric state, but also in kind of π–π aggregated state [93–94]. When more polar solvent MeCN or MeOH were used, merely a broad and very weak absorption band was observed. All the weak and disappearing bands indicate the overlapping of PDI chromophores to form *J*-type aggregates in polar solvents [84]. The intensities of the characteristic absorption bands of **1** in mixed solvents of CHCl_3_/MeCN (v/v = 2:1) are even higher than those of **1** in pure CHCl_3_, indicating a better solubility of **1** in the mixed solvents. We were encouraged to investigate the UV–vis absorption behavior of **1** in mixed solvents of CHCl_3_ and MeCN with different volume ratios (Supporting Information File 1, Figure S9). It is observed that when the volume proportion of MeCN in the mixed solvent is below 70%, the UV–vis absorption intensity of **1** is higher than that in pure CHCl_3_. However, when the volume proportion of MeCN increases to 80%, the UV–vis absorption intensities decrease dramatically. The variations of UV–vis absorbance of **1** in the mixed solvents with different ratios are correlative to its solubility changes, indicating that the solubility of **1** in solvents are probably influenced by the interactions between both PBI unit and crown ether units with solvents. The fluorescence spectra of **1** (λ_ex_ = 490 nm) in different solvents also imply the varied self-assembly behavior (Figure 1b). The fluorescence of **1** in CHCl_3_ is strong with a broad emission band at 520–600 nm, and becomes even stronger when CHCl_3_/MeCN (v/v = 2:1) is used as solvent. But due to the aggregation-caused quenching (ACQ) effect, the fluorescence of **1** in MeCN decreased dramatically, and was almost totally quenched in MeOH and H_2_O. It is surprising to us that the fluorescence of **1** in CHCl_3_ is also quenched even at such a low concentration (5.0 × 10^−6^ M), as only a faint blue fluorescent emission of **1** could be detected (Figure 1b inset). According to previous reports, such a fluorescent quenching indicated the formation of a dimeric structure [67,95], and the electron rich substituents at the imide nitrogen should play an important role [96–97].

For comparison, UV–vis absorption and fluorescence spectra of **2** in the above-tested five solvent systems were also recorded. Because of the relatively weaker π–π stacking effect of the NDI units comparing with PDI units, **2** has a better solubility in the organic solvents. Consequently, the UV–vis absorption bands of **2** in all the organic solvents can be clearly observed but with an overall hypsochromic shift (Figure 1c). The aggregation of **2** also takes place in the solvents with high polarity, such as MeOH and H_2_O, in which **2** shows weak or even no UV–vis absorption. Similar to **1**, **2** also shows a strong fluorescence in CHCl_3_ and mixed CHCl_3_/MeCN (v/v = 2:1, Figure 1d). In the polar solvents MeCN and MeOH, fluorescence of **2** becomes much weaker, and is almost totally quenched in H_2_O.

**Figure 2:**
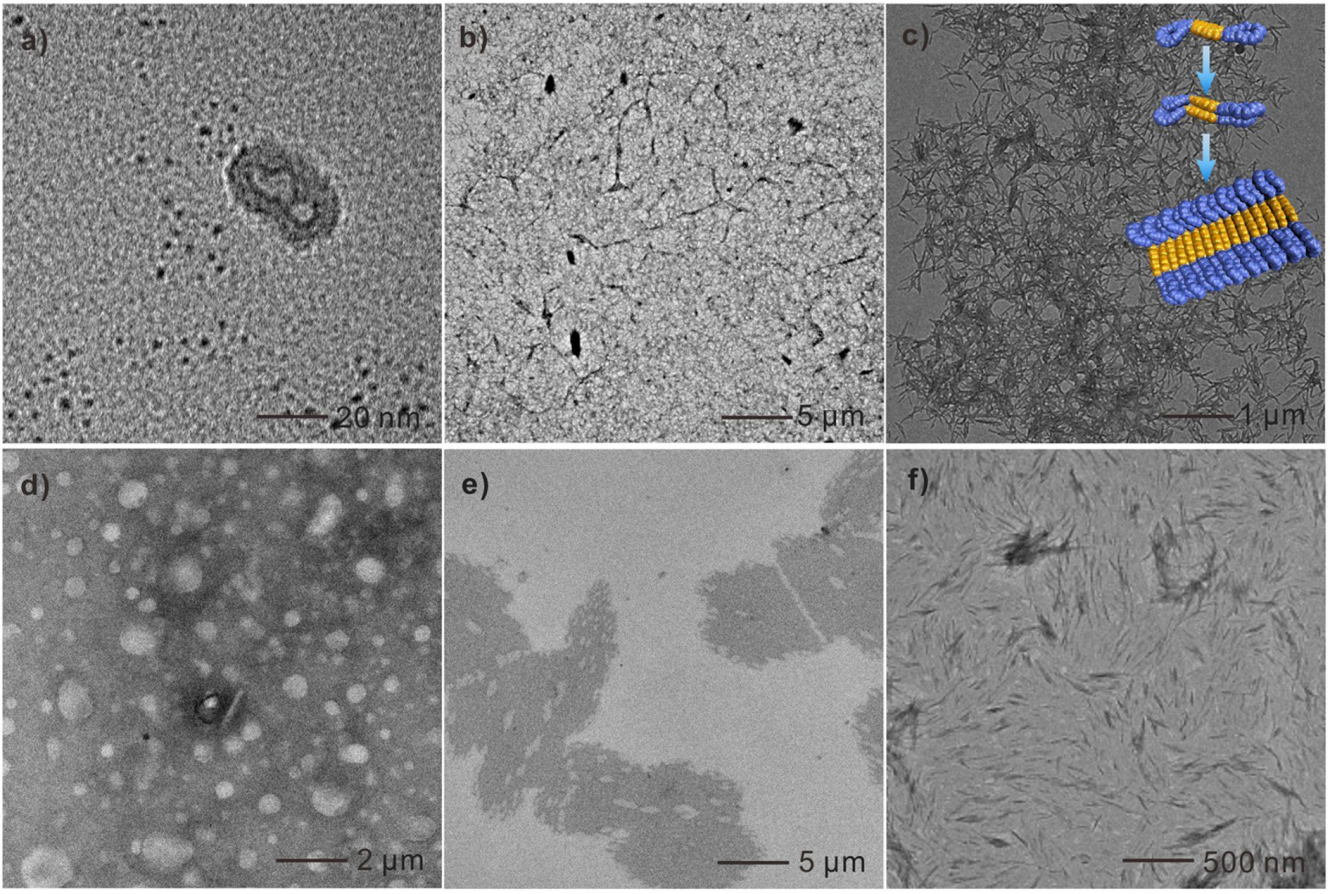
TEM images of **1** aggregates in (a) CHCl_3,_ (b) MeCN, and (c) H_2_O/MeCN (v/v = 10:1) (inset: schematic space-filling models illustrating the self-assembly of **1**); TEM images of **2** aggregates in (d) CHCl_3_, (e) MeCN, and (f) H_2_O/MeCN (v/v = 10:1). Concentration of solutions for all the TEM analysis is 1 × 10^−4^ M.

In order to observe the aggregates formed by **1** and **2** visually in solutions, transmission electron microscopy (TEM) was utilized. First, the aggregates of **1** formed in CHCl_3_ and MeCN were investigated. As shown in Figure 2a, micelle-like assemblies of **1** were observed, while, because of the higher polarity of MeCN, the aggregation of **1** through π–π stacking was considerably enhanced forming linear assemblies (Figure 2b). Interestingly, when imposed to an aqueous medium through adding water slowly into MeCN solution under ultrasonication yielding a mixture solvent of H_2_O/MeCN = 10:1 (v/v), **1** assembled into a well-defined nanobelt in extremely polar aqueous environment, which further entangled into networks (Figure 2c) [68]. Previously, Würthner’s group reported the self-assembly of a trioligo(ethylene glycol)-conjugated PDI system which can self-assemble into similar nanobelt structures [79]. Therefore, we inferred that the B21C7 macrocycle units have a similar solubilizing and induction effect like oligo(ethylene glycol) chains for the self-assembly behavior of PDI derivatives. Due to the weaker π–π stacking of **2** leading to a less regular packing arrangement, no regular morphology was observed when CHCl_3_ or MeCN was used as solvent (Figure 2d and 2e), and only a short nanobelt was observed in H_2_O/MeCN (v/v = 10:1, Figure 2f). The TEM characterization result was corroborated well by a dynamic light scattering (DLS) measurement (Figure S10, Supporting Information File 1), which was proven to be a convenient method to study aggregation behavior in solvents [98–99]. It was found that the average diameter of aggregates of **1** in CHCl_3_ (100 μM) was around 31.1 nm. The size of aggregated **1** in MeCN was ranging from around 40 to 200 nm centered at 81.8 nm, while it further increased to 840.1 nm in H_2_O/MeCN (v/v = 10:1). However, the DLS measurement results of aggregates formed by **2** in either CHCl_3_ or MeCN was unrepeatable, possibly due to the irregular packing in these solvents. But DLS measurement result showed that the size of aggregates of **2** formed in H_2_O/MeCN (v/v = 10:1) was centered at 576.8 nm. It should be noted that the aggregates formed by **1** and **2** with specific shapes were obtained immediately after the preparation of solutions, without days’ evolution process [77].

## Conclusion

In summary, we synthesized crown ether-functionalized PDI and NDI derivatives, and investigated their solvent-dependent solubility and self-assembly behaviors. It was found that the polarity of solvents have an important impact on their self-assembly behavior, thus inducing obvious changes in their UV–vis absorption and fluorescence properties. An unexpected fluorescence quenching phenomenon was observed in the PDI-B21C7 macrocycle conjugate in chloroform. Through TEM characterization, it was illustrated that in aqueous medium, both PDI and NDI derivatives containing crown ether units could quickly self-assembly into nanobelt aggregates, during which the crown ether units exhibited the similar solubilizing and induction effect like oligo(ethylene glycol) chains.

## Supporting Information

File 1 Experimental and analytical data.
