# Supplementary material for: Self-assembly behaviors of perylene- and naphthalene-crown macrocycle conjugates in aqueous medium
Source: Beilstein J Org Chem. 2019 Jun 3;15:1203–9. doi: 10.3762/bjoc.15.117 (PMC6604709; doi:10.3762/bjoc.15.117)
Supplement: File 1 — Experimental and analytical data. [file Beilstein_J_Org_Chem-15-1203-s001.pdf]

BEILSTEIN JOURNAL OF ORGANIC CHEMISTRY

Supporting Information

for

Self-assembly behaviors of perylene- and naphthalene-crown macrocycle conjugates in aqueous medium

Xin Shen, Bo Li, Tiezheng Pan, Jianfeng Wu, Yangxin Wang, Jie Shang, Yan Ge, Lin Jin and Zhenhui Qi

Beilstein J. Org. Chem. **2019,** *15,* 1203–1209. doi:10.3762/bjoc.15.117

Experimental and analytical data

License and Terms: This is a supporting information file under the terms of the Creative Commons Attribution License (http://creativecommons.org/licenses/by/4.0). Please note that the reuse, redistribution and reproduction in particular requires that the authors and source are credited.
The license is subject to the *Beilstein Journal of Organic Chemistry* terms and conditions: (https://www.beilstein-journals.org/bjoc)

General Methods and Materials

All reagents were commercially available and used without further purification. Milli-Q water was used in all measurements. Nuclear magnetic resonance (NMR) spectra were recorded with a Bruker Avance III-400 spectrophotometer, using the deuterated solvent as the lock and residual solvent or TMS as the internal reference. For the structural characterization of synthetic compounds, high-resolution mass spectrometry (HR-MS) was performed using an electrospray ionization (ESI) interface on an Agilent LC/MSD TOF system. Dynamic light scattering (DLS) characterization was performed on Zetasizer Nano ZS, Malvern Instruments, Worcestershire, U. K.

UV and fluorescence spectrometry. The sample was dissolved in different solvents at a concentration of 5 μM. The UV–vis spectra were recorded on a ThermoEvolution 260 Bio UV/Vis/NIR spectrometer with a temperature controllable system in the scan range of 300 to 750 nm using a 1.0 cm quartz micro cuvette. Fluorescence experiments were performed in a 1.0 cm quartz cuvette and recorded on an F-380 spectrofluorimeter (GANGDONG SCI. & TECH.) with 5 nm slit.

Transmission electron microscopy (TEM) images. Droplets (≈5 μL) of sample solution were placed on carbon-film-coated copper grids (ELECTRON MICROSCOPY CHINA) at ambient temperature. The grids were allowed to air-dry for at least 40 min and were subsequently transferred into the microscope without use of a contrasting or cryo-fixation step. TEM images were obtained with a FEI Talos F200X transmission electron microscope with an accelerating voltage of 300 kV.

Synthesis and characterization of compounds

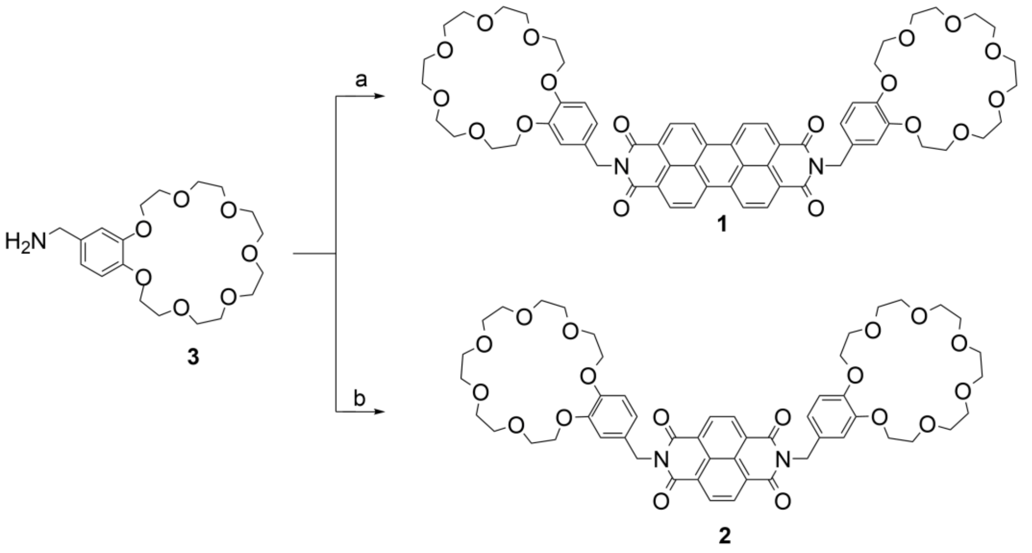

Scheme S1: The synthetic route of compounds **1** and **2**. a) 3,4,9,10-Perylenetetracarboxylic dianhydride (PDI), DMAP, 140 °C; b) 1,4,5,8-Naphthalenetetracarboxylic dianhydride (NDI), DMAP, 140 °C.

Preparation of Compound 3

The synthesis of compound **3** was based on previously reported reference.[1] ^{1}H NMR (400 MHz, $CDCl_3$) δ = 6.84 (s, 3H), 4.15 (s, 4H), 3.91 (d, J = 3.5 Hz, 4H), 3.79 (d, J = 4.9 Hz, 6H), 3.73 (d, J = 4.1 Hz, 4H), 3.67 (s, 8H). HR-MS (ESI) *m/z* (M+H$^+$) *calcd.* for $C_{19}H_{32}NO_7^+$: 386.2179, *Found:* 386.2191.

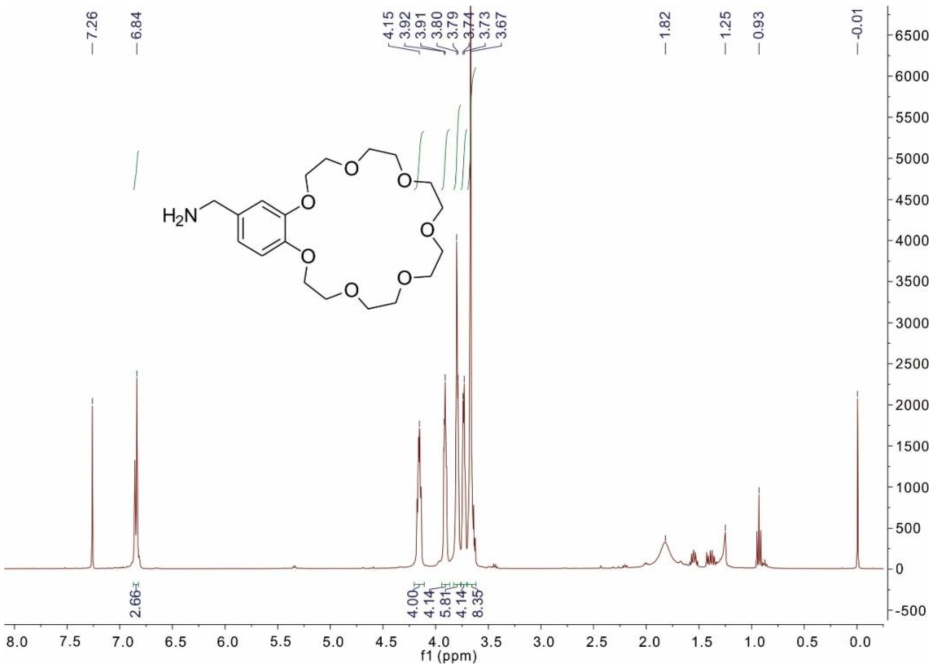

Figure S1: ^{1}H NMR spectrum (400 MHz, $CDCl_3$, 298 K) of **3**.

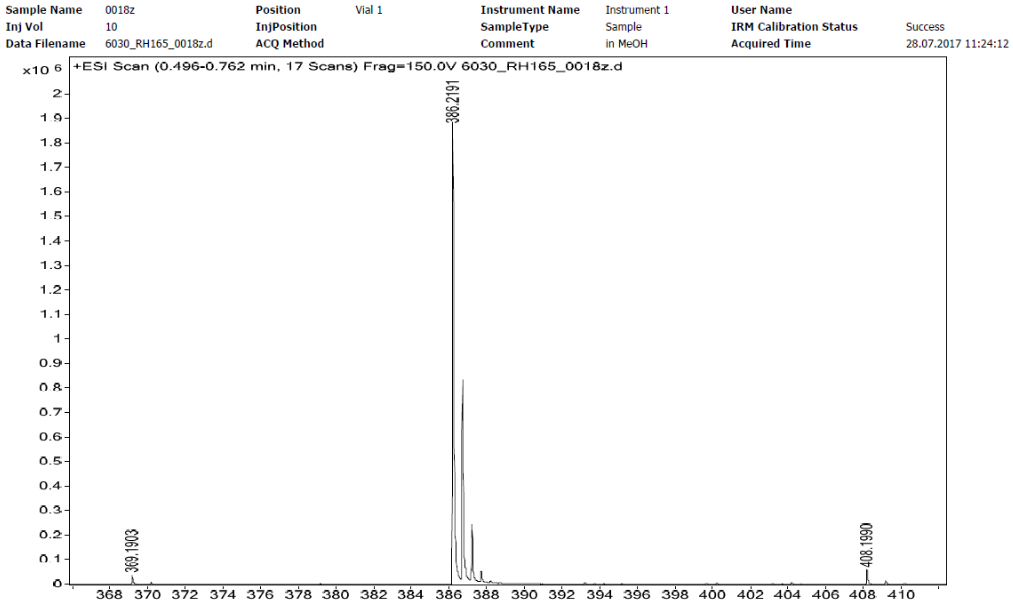

Figure S2: HR-MS characterization of **3**.

Preparation of Compound 1

To the solution of compound **3** (1.03 g, 2.68 mmol) dissolved in glycol (20 mL) in a 50 mL flask, 3,4,9,10-perylenetetracarboxylic dianhydride (0.5 g, 1.28 mmol) and DMAP (7.7 mg, 6.3×10^{-2} mmol) was added. The mixture was heated to 140 °C and stirred for 24 h. After the reaction mass was cooled to room temperature, it was poured into CH_2Cl_2 (200 mL). The organic phase was

washed with HCl aqueous (0.05 M, 100 mL × 2), and brine (100 mL), respectively. Then, the organic layer was dried by Na_2SO_4 and concentrated under reduced pressure to obtain the crude product. The crude product was purified by silica gel column chromatography with MeOH/CH_2Cl_2 = 1/100 to 1/30 (v/v) as eluent to yield the pure product as a brown solid (yield: 1.21 g, 84%). ^{1}H NMR (400 MHz, $CDCl_3$) δ = 8.58 (d, J = 8.0, 4H), 8.43 (d, J = 8.1, 4H), 7.21 (d, J = 9.2, 4H), 6.87 (d, J = 8.1, 2H), 5.33 (s, 4H), 4.28 – 4.19 (m, 4H), 4.19 – 4.10 (m, 4H), 3.99 – 3.86 (m, 8H), 3.80 (t, J = 6.5, 3.1, 8H), 3.73 (t, J = 6.4, 3.3, 8H), 3.66 (s, 16H). ^{13}C NMR (100 MHz, $CDCl_3$) δ = 163.22, 148.77, 148.50, 134.43, 131.44, 130.31, 129.14, 126.16, 123.17, 122.99, 122.69, 115.67, 113.99, 71.13, 71.08, 71.01, 70.98, 70.54, 69.78, 69.73, 69.35, 43.41. HR-MS (ESI) *m/z* (M+Na$^+$) *calcd.* for $C_{62}H_{66}N_2O_{18}Na^+$: 1149.4208, *Found:* 1149.4218; *m/z* (M+K$^+$) *calcd.* for $C_{62}H_{66}N_2O_{18}K^+$: 1165.3948, *Found:* 1165.3960.

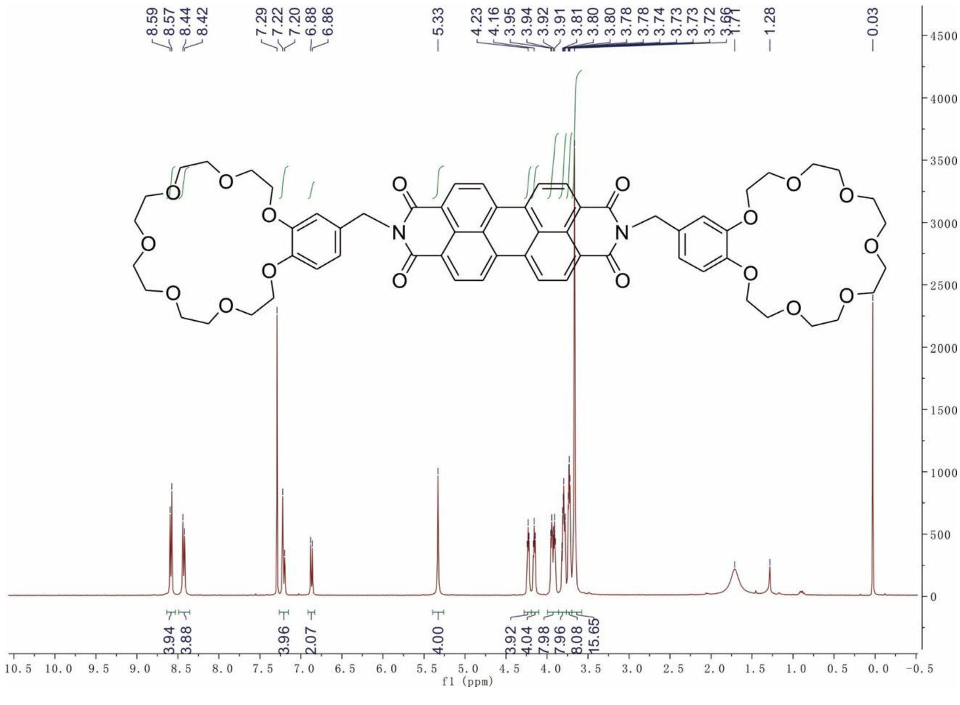

Figure S3: ^{1}H NMR spectrum (400 MHz, $CDCl_3$, 298 K) of **1**.

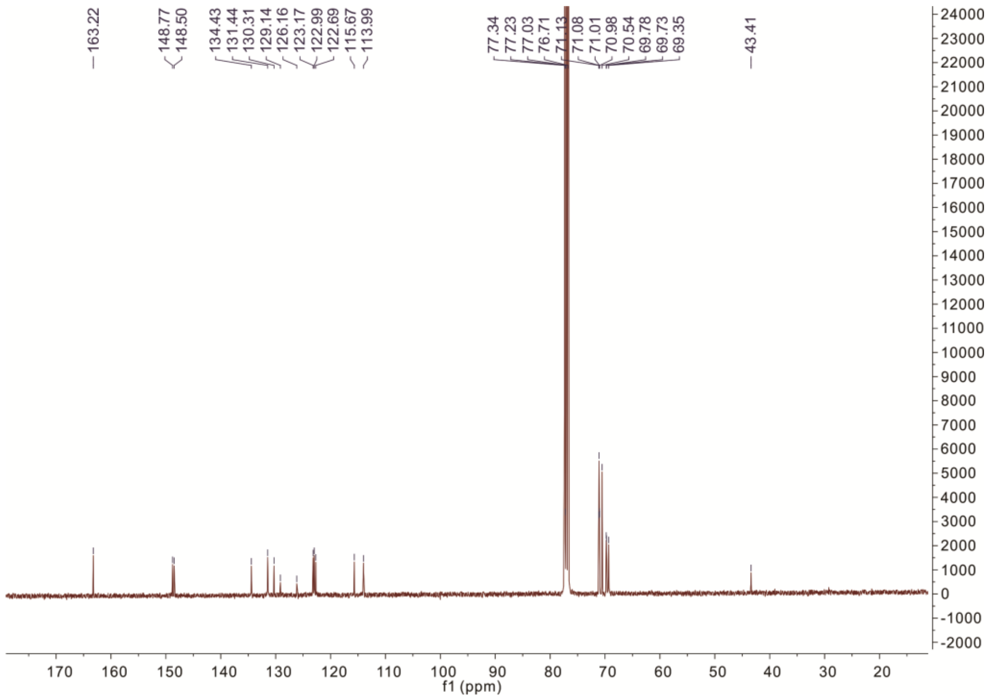

Figure S4: ^{13}C NMR spectrum (100 MHz, $CDCl_3$, 298 K) of **1**.

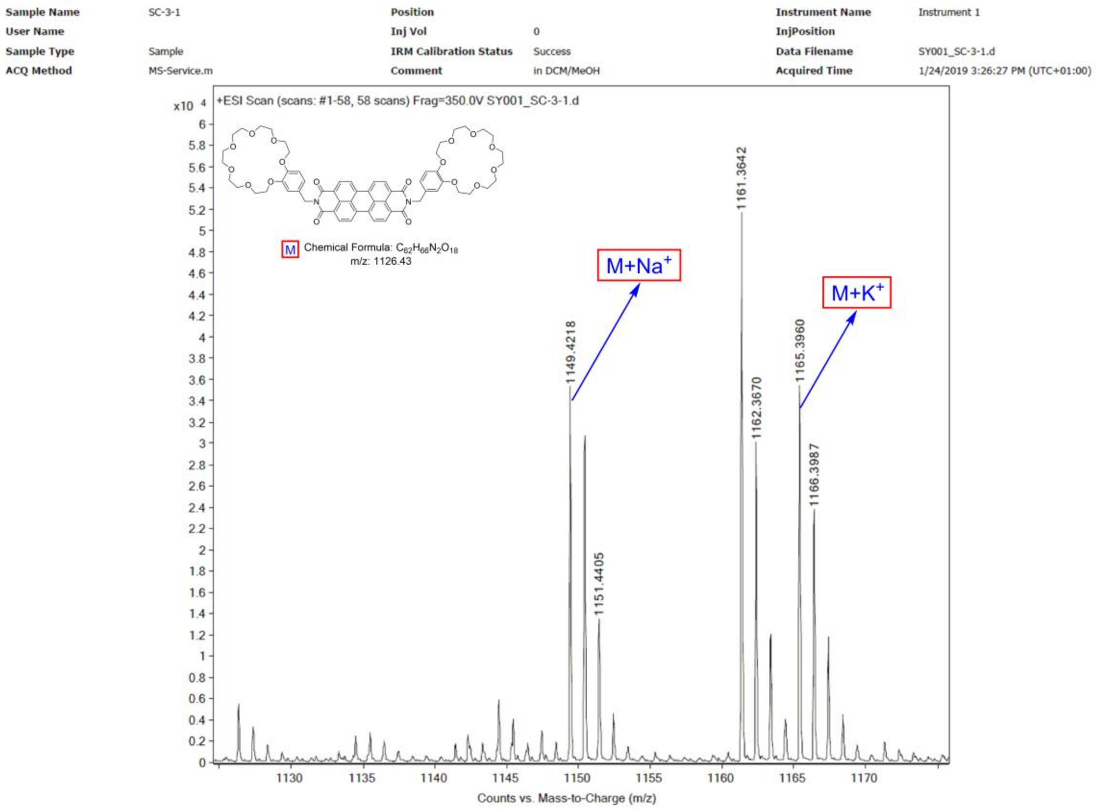

Figure S5: HR-MS characterization of **1**.

Preparation of Compound 2

To the solution of compound **3** (0.75 g, 1.95 mmol) dissolved in 20 mL glycol in a 50 mL flask, 1,4,5,8-naphthalenetetracarboxylic dianhydride (0.25 g, 0.93 mmol) and DMAP (5.7 mg, 4.6×10^{-2} mmol.) were added. The mixture was heated to 140 °C and stirred overnight. After the reaction mass was cooled to room temperature, it was poured into CH_2Cl_2 (100 mL). The organic phase was washed by HCl aqueous (0.05 M, 50 mL × 2) and brine (50 mL), respectively. Then, the organic layer was dried by Na_2SO_4 and concentrated under reduce pressure to obtain the crude product. The crude product was purified by silica gel column chromatography with $MeOH/CH_2Cl_2$ = 1/100 to 1/30 (*v*/*v*) as eluent to yield pure product as yellow solid (yield: 0.71 g 76%). ^{1}H NMR (400 MHz, $CDCl_3$) δ = 8.78 (s, 4H), 7.16 (d, *J* = 7.3, 4H), 6.84 (d, *J* = 8.5, 2H), 5.32 (d, *J* = 2.5, 4H), 4.23 – 4.16 (m, 4H), 4.16 – 4.11 (m, 4H), 3.97 – 3.86 (m, 8H), 3.83 – 3.75 (m, 8H), 3.75 – 3.70 (m, 8H), 3.70 – 3.59 (m, 16H). ^{13}C NMR (100 MHz, $CDCl_3$) δ = 162.83, 148.75, 148.59, 131.13, 129.72, 126.69, 122.68, 115.61, 113.91, 71.09, 71.06, 70.99, 70.97, 70.54, 69.73, 69.68, 69.27, 69.24, 43.69. HR-MS (ESI) *m/z* ($M+Na^+$) *calcd.* for $C_{52}H_{62}N_2O_{18}Na^+$: 1025.3895, *Found:* 1025.3896；*m/z* ($M+K^+$) *calcd.* for $C_{52}H_{62}N_2O_{18}Na^+$: 1041.3635, *Found:* 1041.3633.

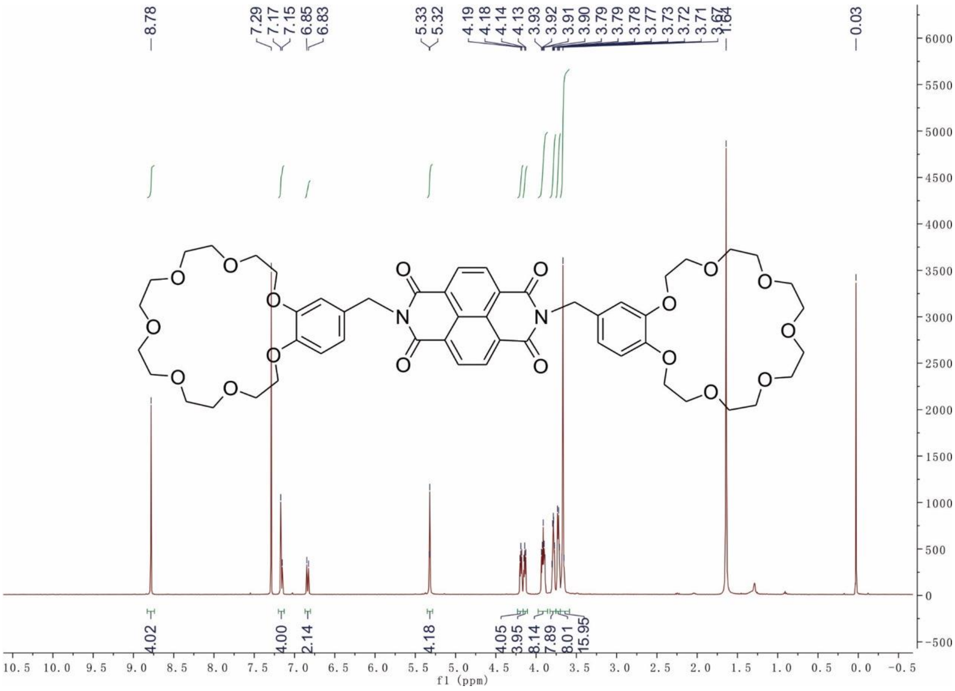

Figure S6: ^{1}H NMR spectrum (400 MHz, $CDCl_3$, 298 K) of **2**.

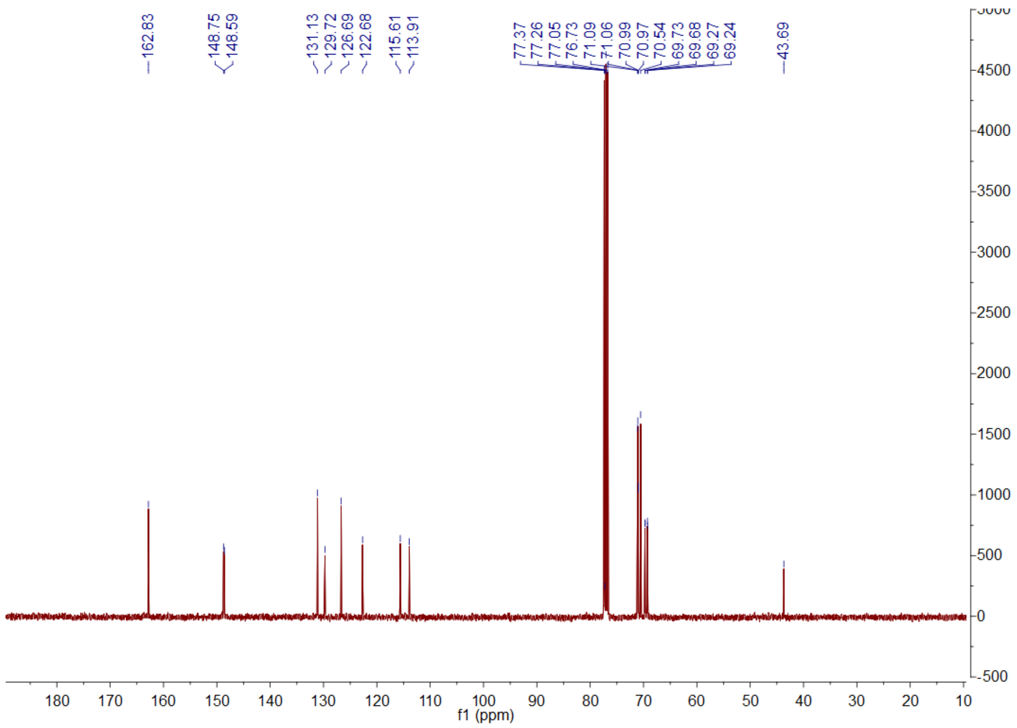

Figure S7: ^{13}C NMR spectrum (100 MHz, $CDCl_3$, 298 K) of **2**.

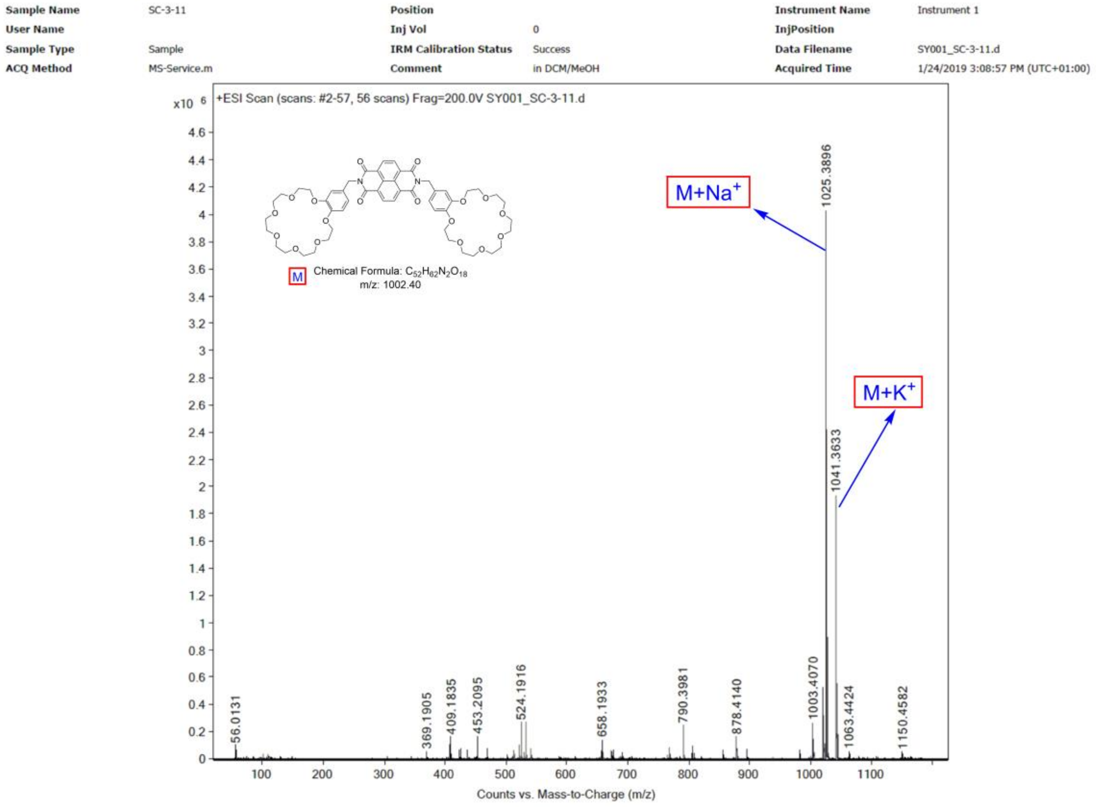

Figure S8: HR-MS characterization of **2**.

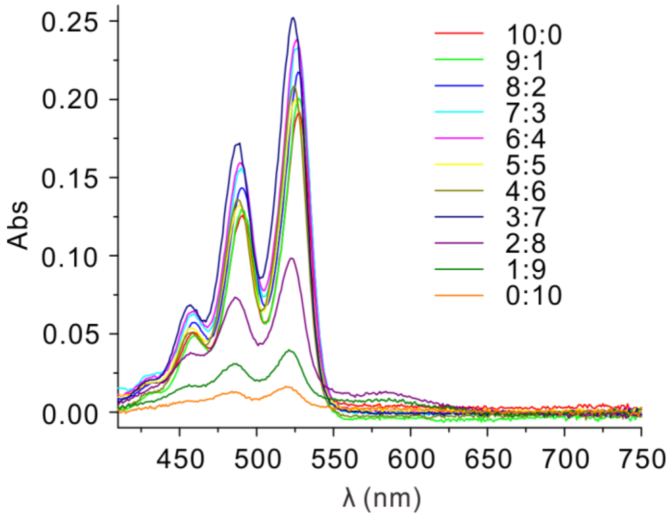

Figure S9: UV–vis spectra of **1** in (mixed) solvents of $CHCl_3$ and MeCN with different volume ratio ranging from 10:0 to 0:10.

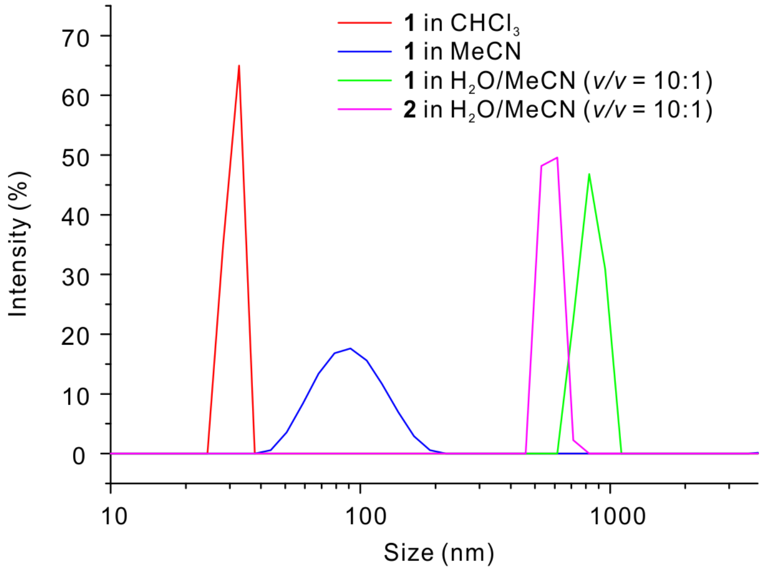

Figure S10: Size distribution of aggregates formed by **1** and **2** in different solvents (100 µM, 25 °C) based on DLS measurements.

Reference

1. Qi, Z.; de Molina, P. M.; Jiang, W.; Wang, Q.; Nowosinski, K.; Schulz, A.; Gradzielski, M.; Schalley, C. A. *Chem. Sci.*, **2012**, *3*, 2073-2082.
